# Mismatch negativity in children with dyslexia speaking Indian languages

**DOI:** 10.1186/1744-9081-3-36

**Published:** 2007-07-31

**Authors:** Vanaja Chittinahalli Shankarnarayan, Sandeep Maruthy

**Affiliations:** 1 Department of Audiology, All India Institute of Speech and Hearing, Mysore, India

## Abstract

**Background:**

Several studies in the past have found that phonological processing is abnormal in children with dyslexia. Phonological processing depends on the phonological rules of the language learnt. Western languages do not have a good phoneme to grapheme correspondence while many of the Indian languages do have it. Also phonological rules of western languages are different from that of Indian languages. Thus it would be erroneous to generalize the results of phonological processing obtained on children speaking western languages to those speaking Indian languages. Hence the present study was aimed to investigate the auditory processing in children with dyslexia who spoke and studied Indian languages.

**Methods:**

Standard group comparison design was used in the study. The study was conducted on fifteen children with dyslexia and fifteen control children. Mismatch negativity was elicited for speech and tonal stimuli. Results obtained on the clinical group were compared with that of control group using mixed design Analysis of variance. Children in both the groups were native speakers of Kannada (a south Indian language).

**Results:**

A subgroup of children showed abnormalities in the processing of speech and/or tonal stimuli. Speech elicited mismatch negativity showed greater abnormalities than that of tonal stimuli. Though higher for spectral contrasts, processing deficits were also shown for durational contrasts.

**Conclusion:**

Inspite of having different phonological rules and good phoneme-grapheme correspondence in Indian languages, children with dyslexia do have deficits in processing both spectral and durational cues.

## Background

Developmental dyslexia is a specific disability in learning to read and spell adequately despite at least normal intelligence, adequate instruction, adequate socio-cultural opportunities, and the absence of sensory defects in vision and hearing [1]. In India, prevalence of dyslexia varies between 3% and 10% [2].

The causes of dyslexia are numerous and often are poorly defined. There are several theories that attempt to account for dyslexia. Snowling [3] classifies the theories that have received most attention into two general approaches. The first is domain specific view, which posits that the dyslexia arise from deficits in systems that are specifically linguistic; in phonological processing and memory. The second school of thought claims the core deficits in underlying nonlinguistic sensory mechanisms, which could be either in visual processing or in auditory processing.

In the last two decades, studies have explored a close link between phonological skills and reading. Torgesen [4] found poor phonological awareness in children with dyslexia. The primary deficit in developmental dyslexia in all languages has been reported to be in representing speech sounds as graphemes [5]. Furthermore, intervening at the level of phonological skills has been found to improve reading outcomes for children who struggle to read their first language [6-8]. Vandervelden and Siegel [9] showed that the phonological intervention helped the low ability group to improve their reading skills, thus highlighting the importance of phonological processing in reading. Children with dyslexia are known to have reading difficulty. A thorough investigation of phonological skills in each of these children may help us to better understand the cause of the disability and to design a better rehabilitative approach.

Phonological discrimination in children with dyslexia has been assessed using both behavioral and electrophysiological tests. Using behavioral discrimination tasks, [10,11], low-level processing deficits that cause problems in discriminating rapid temporal changes is reported. The finding that dyslexics are mainly impaired in processing stop consonants [11], indicate the presence of temporal processing deficit, as stop consonants are characterized by brief and rapid spectral changes. If such impairment exists, it may disturb the adequate development of phonological codes from an early age.

Most of the electrophysiological studies carried out to investigate phonological discrimination have used MMN. MMN reflects automatic, pre attentive auditory discrimination, represented as a small negative deflection superimposed on N_1_P_2 _or P_2_N_2 _complex [12]. Neural activity within MMN may reflect early cognitive processes in terms of pre-attentive comparison of an incoming deviant stimulus with a stored memory trace of the preceding standard events [13]. MMN, when compared to other auditory potentials appears to represent a better correlate of language specific phonetic traces that serve as recognition models for speech sound during auditory perception [14].

Schulte-Korne et al. [15] elicited MMN for syllable (/ba-da/) and tonal (1000 Hz–1050 Hz) deviances and, compared the results between children with dyslexia and controls. Results showed that the MMN elicited by the syllable deviance was diminished in subjects with dyslexia when compared to that of controls. However, MMN amplitude elicited by the tone deviance did not differ significantly from that of controls. They interpreted their results in terms of a speech-specific pre-attentive processing deficit in children with dyslexia. However, contradictory results which show poor MMN amplitude even for tonal deviance are also reported [16].

Furthermore, within speech the perception of all spectro-temporal changes might not be impaired to the same extent. Kraus et al. [17] compared the performance of normal children and children with learning problems in behavioral discrimination task (/ba-wa/&/da-ga/) as well as MMN. Results showed that children with learning problems had deficit in discrimination of /da-ga/ but showed intact performance in discriminating /ba-wa/. MMN recorded from these subjects correlated with the behavioral findings. MMN elicited with the /da/-/ga/ pair had diminished magnitude and prolonged latency whereas /ba/-/wa/ pair elicited normal MMN. This may be because, two different contrasts may tap into separate and distinct neural mechanisms or they may be processed at distinct locations along the auditory pathway. Furthermore, the presence of MMN does not indicate that the stimuli can be discriminated behaviourally. Bradlow et al. [18] investigated behavioral discrimination of /da/-/ga/ and its neurophysiologic correlate. It was observed that varying the formant transition duration from 40 ms to 80 ms did not result in improved behavioral response but there was enhancement of MMN response.

Thus, the results of both behavioral and electrophysiological methods show the presence abnormal phonological discrimination in children with dyslexia. But, most of the published studies are done on children who had English or other western languages as their native language. These languages are alphabetic and do not have a good phoneme to grapheme correspondence whereas, Indian languages are semi-syllabic in nature and there is almost a one to one grapho-phonological equivalence expressed in syllabic structure with the regular signs of vowels being attached to the basic consonant form [19]. The phonological rules in Indian languages are different from that of English or other western languages. This may influence phonological processing. Hence, these data obtained on children who spoke English or other western languages may not be applicable to children speaking Indian languages. Despite having high prevalence rate of dyslexia in India, studies have not been conducted on children who speak Indian languages. Thus the present study was taken up to investigate MMN in children with dyslexia who spoke and studied Indian languages.

## Methods

The study was conducted on two groups of children. One group was a clinical group and fifteen children with the diagnosis of dyslexia participated in this group. The diagnosis of dyslexia was made by an experienced speech and language pathologist/Clinical psychologist. Profiling of children was done to identify the core features of dyslexia in them.

The second group was a control group and thirty children with age appropriate scholastic performance participated in the experiment. Age of the children in both the groups ranged between 7 years and 12 years. The native language and the medium of instruction was Kannada (A Dravidian Language spoken in south India) for children in both the groups. Pure tone audiometry and immittance evaluation were administered for each child to ensure normal hearing sensitivity and normal middle ear functioning. A detailed case history was taken prior to the experiment to rule out any relevant otological or neurological pathology. Before subjecting the children to the experiment, consent was taken from parents of the children.

### Stimulus parameters

**Figure 1:**
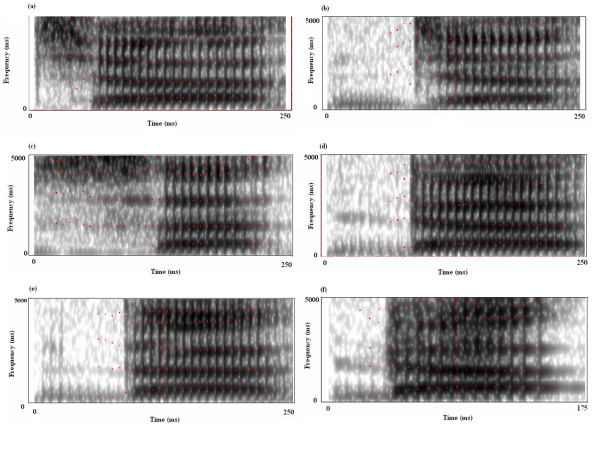
(a to f). The figure shows spectrograms of six syllables used to elicit MMN. The total duration of stimuli /tΣa/, /dZa/, /sa/, /da/&/δ8a/ was approximately 250 ms. Whereas, duration of da_s_/ was 175 ms. All the syllables spoken by an adult male speaker were digitally recorded at a sampling frequency of 16 kHz and 16-bit digitisation. They were then edited using analysis by synthesis method to achieve the target duration and were normalized.

Mismatch negativity was recorded for spectral as well as temporal contrasts using speech and tonal stimuli. Six syllables, /tΣa/ [see Additional file 1], /dZa/ [see Additional file 2], /sa/ [see Additional file 3], /da/ [see Additional file 4], /δ8a/ [see Additional file 5] and /da_s_/ [see Additional file 6] were used to create four syllable contrasts that could elicit MMN. /da_s_/ refers to shorter duration /da/. Place of articulation, manner of articulation, voicing and syllable duration were the four target contrasts. Except /da_s_/, all the syllables were 250 ms in duration. Stimuli were generated using analysis by synthesis method using Praat signal editing software (version. 4.2.01). Syllable /da_s_/ was 175 ms in duration. Stimulus /da/ and /da_s_/ differed only in their vowel duration. All the syllables spoken by an adult male speaker were digitally recorded at a sampling frequency of 16 kHz and 16-bit digitisation. Syllables were edited to improve signal to noise ratio as well as to maintain target the duration. Stimuli were then normalized and loaded into Smart EP system using STIMCONV software. Spectrograms of the stimuli are shown in the figure 1(a–f).

Two separate MMN recordings were done using speech syllables. In the first recording, /tΣa/ stimulus was used as a standard while the /dZa/ and /sa/ were used as deviants, differing in voicing and manner of articulation respectively. In the second recording, /da/ stimulus was used as a standard while /δ8a/ and /da_s_/ were used as deviants, differing in place of articulation and vowel duration respectively.

Tonal stimuli were generated using stimulus generator of Intelligent Hearing Systems, Smart EP (version. 3.70). A 1000 Hz puretone of 250 ms duration was used as standard while a 1100 Hz puretone of 250 ms and 1000 Hz puretone of 175 ms were deviants, deviating in frequency and duration respectively. As the acoustical differences in the place, manner, voicing and syllable duration are either in terms of frequency or duration, for comparison with the MMN elicited by syllable, frequency and duration deviances were taken up in the tonal contrasts. The stimuli were presented binaurally at 70 dB nHL through ER3A insert earphones.

### Recording parameters

The potentials elicited by the test stimuli were recorded and analysed using Intelligent Hearing Systems, Smart EP (version. 3.70). The responses were differentially recorded from F_Z_, T_L _and T_R _(positive) with reference to nose tip (negative) using silver chloride electrodes. T_L_was located half way between T_3 _and T_5 _and, T_R _was located half way between T_4 _and T_6_. The ground electrode was placed on nasion. The responses were averaged online for a block of 100 sweeps where each sweep consists of a deviant stimulus and a set of standard stimuli. The probability ratio of standard stimulus and deviant stimulus was 5:1. Thus, the total number of standard stimuli presented was around 500. The repetition rate was kept constant at 1.9 stimuli per second. The analysis window was set to 500 ms. The EEG was band pass filtered online between 1 Hz and 30 Hz. The first two sweeps in each block were discarded during the recording, as they elicit considerably larger exogenous responses than the following ones [20]. Any block with artifacts exceeding 5% of the total sweeps was not considered for the analysis.

### Test procedure

Children sat in a comfortable position to ensure a relaxed posture and minimum muscular artefacts. During the recording, children watched a silent cartoon movie of their choice. This probably minimized the possibility of active attention and reduced the eye blinking, both of which could affect the MMN recording. Before starting the recording, low absolute (<5 kΩ) and relative (<2 kΩ) electrode impedance was ensured. Stimuli were presented binaurally in an oddball paradigm.

### Analysis

MMN was determined by subtracting the averaged waveform for standard stimulus from the averaged waveform for deviant stimulus. In the difference wave, the first negative peak after N_1 _(in the deviant wave), with greater than 0.5 μV negative amplitude, was identified as MMN. Four experienced audiologists independently analysed the difference waveforms to identify the MMN. It was considered as a response only if all the four audiologists identified the MMN at the same latency. Peak latency and peak amplitude of the MMN was noted for each waveform. The data obtained from the control group and clinical group was statistically analysed using Mixed design ANOVA.

## Results

### Latency of MMN

**Figure 2:**
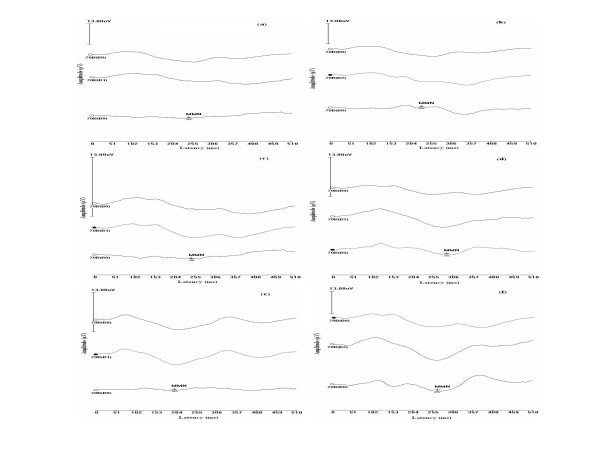
(a to f). The figure shows grand mean MMNs of control group. Figure 2a to 2f represents the waves elicited for /tΣa/-/dZa/, /tΣa/-/sa/, /da/-/δ8a/, /da/-/da_s_/, tonal frequency and tonal duration contrasts respectively. Each sub-figure has a standard, a deviant and a difference wave respectively. The waves are scaled to 13 μV and the MMN is marked on the difference wave.

**Figure 3:**
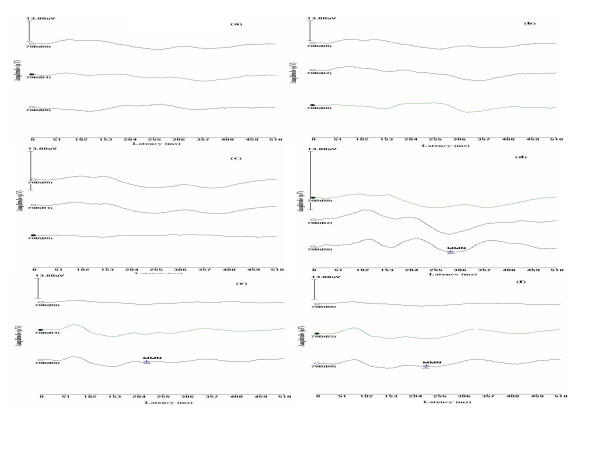
(a to f). The figure shows grand mean MMNs of clinical group. Figure 2a to 2f represents the waves elicited for /tΣa/-/dZa/, /tΣa/-/sa/, /da/-/δ8a/, /da/-/da_s_/, tonal frequency and tonal duration contrasts respectively. Each sub-figure has a standard, a deviant and a difference wave respectively. The waves are scaled to 13 μV and the MMN is marked on the difference wave.

The grand average of standard, deviant and the difference waveforms, recorded from Fz for control and the clinical group children are shown in Fig 2 and 3 respectively. Table 1 shows the mean and standard deviation of peak latency of MMN obtained from control and clinical groups, for the 6 deviances used in the study. For all the deviances, the mean latency of MMN was longer in the clinical group when compared to that of normal group. This was true for MMN picked up from all the electrode sites. In the control group, for speech stimuli, the peak latency was longer at F_Z _followed by that at T_R _and T_L _sites. Such clear trends were not seen for the clinical group. But for tonal stimuli, latency was shortest at F_Z _both in control and clinical groups. Comparison of the latency of MMN for different stimuli shows that the latencies were shorter for tonal stimuli than that for speech stimuli in both control and clinical groups.

**Table 1:** Mean and standard deviation of peak latency (in ms) of MMN

**Stimuli**		**Group**	**F_Z_**		**T_L_**		**T_R_**	
			Mean	SD	Mean	SD	Mean	SD

**Speech**	/tΣa/-/dZa/	Control	225.83	37.23	216.58	48.66	221.64	47.52
		Clinical	296.43	46.36	304.78	47.23	309.0	51.38
	/tΣa/-/sa/	Control	237.86	48.25	219.06	52.27	224.80	48.20
		Clinical	288.75	90.65	293.4	34.46	274.25	28.00
	/da/-/δ8a/	Control	237.47	24.63	212.6	35.62	213.90	38.23
		Clinical	277.5	41.03	290.4	45.29	293.56	41.56
	/da/-/da_s_/	Control	254.87	20.67	218.67	50.17	225.6	52.58
		Clinical	267.85	38.25	281.0	40.56	284.85	41.73

**Tone**	1000 Hz–1100 Hz	Control	207.5	15.43	209.56	25.05	213.37	25.76
		Clinical	212.06	52.9	248.38	77.41	254.92	83.04
	250 ms–175 ms	Control	208.27	20.94	217.27	28.9	219.47	32.96
		Clinical	223.0	61.94	243.21	68.82	249.79	75.61

Mixed design ANOVA was carried out to investigate the effect of group, electrode site and the deviance used on latency of MMN. As the data violated assumption of sphericity, Greenhouse-Gieser effect was used to correct the results. Results revealed that there was a main effect of group [F(1,14) = 31.65, p < 0.01] but repeated measures showed that electrode site [F(1,14) = 0.81, p > 0.05] and stimuli [F(1,14) = 0.65, p > 0.05] did not affect the results. There was a significant interaction between site and stimuli [F(1,14) = 4.54, p < 0.01], site and group [F(1,14) = 3.84, p < 0.05] as well as group, site and stimuli [F(1,14) = 4.5, p < 0.01]. The results of ANOVA thus revealed that latencies of MMN in children with learning disability were significantly longer than those observed in the control group. However, the effect was not similar for different stimuli and at different electrode locations. Therefore Independent t test was carried out to investigate the significant difference between groups for latency of MMN picked up from different sites for different stimuli. The results shown in Table 2 indicate that the latency of MMN was significantly longer in the experimental group when compared to that of control group, for responses picked up form all the sites when the /tΣa/-/dZa/ and /da/-/δ8a/ contrasts. For deviances, /tΣa/-/sa/ and /da/-/da_s_/ contrasts as well as for frequency deviance, the experimental group had significantly longer latency for responses picked form temporal regions but there was no significant difference between the responses picked up from forehead. There was no significant difference between the two groups for duration deviance. Figure 2(a–f) and figure 3(a–f) show the grand average waves for the 6 stimulus contrasts in control and clinical groups respectively.

**Table 2:** t values for MMN for different deviances

	**Stimulus contrasts**					
**Electrode site**	/tΣa/-/dZa/	/tΣa/-/sa/	/da/-/δ8a/	/da/-/da_s_/	1000 Hz–1100 Hz	250 ms–175 ms

Fz	-4.29*	-1.77	-3.74*	-1.47	-.44	-1.19
TL	-4.09*	-2.64*	-5.88*	-4.05*	-2.5*	-1.77
TR	-3.86*	-1.56	-5.38*	-3.7*	-2.51*	-1.87

* significant at 0.01 level

### Amplitude of MMN

The mean and standard deviation of MMN amplitude at 3 electrode sites, for 6 contrasts are shown separately for control and clinical groups in Table 3. Results of mixed design ANOVA indicate that there was no effect of group [F(1,14) = 0.18, p > 0.05], stimuli [F(1,14) = 0.48, p > 0.05], electrode site [F (1,14) = 2.44, p > 0.05] on peak amplitude of MMN. As in the previous analysis, the data violated assumption of sphericity and hence, Greenhoouse-Geiser estimate was used to correct the results. Two way interaction between the variables were not observed but there was a significant three way interaction among the effect of electrode site, stimuli and group [F(1,14) = 2.16, p < 0.05].

**Table 3:** Peak amplitude (μV) of MMN in control and clinical groups

**Stimuli**		**Group**	**F_Z_**		**T_L_**		**T_R_**	
			Mean	SD	Mean	SD	Mean	SD

**Speech**	/tΣa/-/dZa/	Control	-2.2	1.67	-2.4	1.73	-2.78	1.93
		Clinical	-4.12	3.25	-4.31	1.77	-4.55	3.19
	/tΣa/-/sa/	Control	-3.44	2.5	-3.52	2.29	-3.42	2.33
		Clinical	-2.36	1.97	-3.97	1.68	-3.48	2.12
	/da/-/δ8a/	Control	-2.77	2.13	-3.07	1.97	-2.82	2.11
		Clinical	-2.22	1.73	-3.15	1.54	-2.38	1.47
	/da/-/da_s_/	Control	-5.54	3.05	-4.18	2.3	-3.29	2.43
		Clinical	-5.39	3.28	-4.01	1.77	-4.25	2.38

**Tone**	1000 Hz–1100 Hz	Control	-2.68	1.7	-2.89	2.29	-2.73	2.07
		Clinical	-4.08	2.96	-4.41	3.95	-2.77	5.41
	250 msec–175 msec	Control	-2.48	1.57	-2.98	2.17	-3.18	2.42
		Clinical	-4.85	2.77	-4.07	2.08	-3.9	2.5

### Analysis of individual data

The individual data of each subject was analysed to check whether the latency and amplitude values fall within **mean + 1 SD **of that of control group. Such responses which were beyond 1 SD were termed prolonged responses. Table 4, shows the number of children with dyslexia who had normal MMN, prolonged MMN latency or absent MMN.

**Table 4:** The number of children with dyslexia having normal, prolonged and absent MMN.

	**MMN**		
	
**Stimulus contrast**	Normal	Prolonged	Absent
/tΣa/-/dZa/	3	4	8
/tΣa/-/sa/	5	5	5
/da/-/δ8a/	6	4	5
/da/-/da_s_/	5	7	3
Frequency deviance	9	4	2
Duration deviance	9	4	2

## Discussion

Controversy exists regarding the underlying cause of dyslexia. Some investigators assume that the deficit in auditory processing is the source of the phonological disorder observed in children with dyslexia. Others maintain that the phonological deficit in dyslexia is basically linguistic, not acoustic in nature. It is widely accepted that most children with developmental dyslexia perform poorly on tasks that assess phonological awareness. According to one school of thought, the "input" phonological representations of speech sounds are distorted or noisy in these children and this leads to phonological problems [21]. The results of present study support this hypothesis. However, it also shows that not all children with dyslexia have auditory processing problem. There is a subgroup of children who have auditory processing problem though it cannot be ascertained whether the auditory processing problem is the causal factor for dyslexia or it is just an associated factor.

Earlier investigations have proved that many features like voice onset time and formant transitions require the detection of fine timing differences (in few milliseconds) of complex auditory patterns and this is affected in subjects with dyslexia [22]. There is a controversy as to whether the processing of both non speech and speech signal are affected or processing of only speech signal is affected. The main finding of this study was that, the children with dyslexia show impaired auditory processing at the cortical level and the abnormality varies with the stimuli used. Though more number of children showed abnormality in the speech elicited MMN, processing of both speech and tonal signal was affected in children with dyslexia. From the results of the present study, it can be inferred that the discrimination of signals depends on the cues used for processing the signal and not on whether it is speech or non-speech stimuli.

### MMN for speech stimuli

The type of deviances used in the present study included deviances in terms of place of articulation, manner of articulation, voicing and vowel duration. Both behavioral as well as electrophysiological studies have earlier reported that processing of cues for perception of place of articulation is affected in these children [15,17,23]. Kraus et al. [17] reported that behavioral speech sound discrimination and MMN for /ba/-/wa/ continuum was not affected in children with dyslexia while /da/-/ga/ continuum was affected. Schulte-Korne et al [15] observed that the MMN for /ba/-/da/ contrast was affected in children with dyslexia. These results indicate that the features that signal place of articulation appear to be particularly vulnerable when auditory processing breaks down.

There is a dearth for information on MMN for voicing contrast in children with dyslexia. Manis et al. [24] reported that dyslexics with low phonemic awareness made poorer /b/-/p/ distinctions than both chronological age matched and reading level matched controls. They concluded that some dyslexic children have a perceptual deficit that may interfere with processing of phonological information. In the present study, the clinical had significantly higher latency than that of control group irrespective of the stimuli used. However, analysis of the individual data showed that although 9 out of 15 children were abnormal in the processing of /da/-/δ8a/ contrast, more number of children had abnormal processing of /tΣa/-/dZa/ contrast indicating that even processing of durational cues are affected in children with dyslexia. This could be due to the difference in the phonological rules between English and Indian languages and/or the difference in the stimuli used. The present study used natural stimuli and though the major cue used for differentiating /tΣa/-/dZa/ contrast is voice onset time, spectral differences also would have contributed for the discrimination. The F1, F2, F3 differences between the two syllables were 45 Hz, 24 Hz and 38 Hz, respectively.

The deficit in the processing of duration cues was also reported by Leppanen et al. [25]. He reported that processing of durational cues is affected in infants at risk for dyslexia due to a familial background of reading problems. They process auditory temporal cues of speech sounds differently from infants without such a risk even before they learn to speak. In the present study also, speech contrasts which differed in duration elicited abnormal MMN suggesting deficient perception of durational cues in a subgroup of children with dyslexia. The results of the present study support these findings and also reveal that there is a neurophysiologic basis for the abnormal voiced-voiceless perception, observed in these children. Temporal processing deficit in children who spoke and studied Indian languages is further supported by abnormal processing of /da/-/da_s_/ contrast. However, the severity of deficit was higher for the processing of /tΣa/-/dZa/ than that of /da/-/da_s_/. This could be because, the VOT difference in /tΣa/-/dZa/ contrast was 25 ms whereas the vowel duration differed by 75 ms in /da/-/da_s_/ contrast.

### MMN for tonal stimuli

The results of the present study and the review of literature suggest that the processing of speech syllables is affected in children with dyslexia. However, there is a controversy as to whether this type of deficit exists for tonal stimuli. The results of the present study show that these children have abnormal MMN for tonal stimuli also. When tonal stimuli were used to record MMN, no group difference was obtained for MMN for duration deviation whereas MMN for frequency deviance showed a group difference. Earlier investigation by Schulte-Korne et al. [15] showed that MMN for frequency deviance of tone was not affected in dyslexics whereas MMN for speech stimuli was affected. They had concluded that dyslexics have a specific speech processing deficit at the sensory level which could be used to identify children at risk at an early age. Similar results have also been reported in adults with dyslexia [26]. However, Baldeweg et al. [16] reported that even MMN elicited for tonal contrasts were smaller in dyslexics. An investigation by Schulte-Korne et al. (1999) using complex tonal pattern that differed in temporal pattern but not frequency, showed that dyslexics have a significant pre-attentive deficit in processing of rapid temporal patterns. Renvall and Hari [27] reported that electroencephalographic studies demonstrate smaller auditory responses to infrequent deviances of speech and non speech sounds in dyslexic than normal-reading subjects. The results of the present study reinforces the consensus that processing of both speech and non speech stimuli is affected in children with dyslexia but processing of all the cues are not equally affected.

Based on the results of the present study and earlier investigations, it can be inferred that it may be the acoustic information embedded in speech sounds, rather than phonetic information per se, that resulted in the attenuated MMN found in dyslexics. Behavioral studies reported in literature also indicate that processing of both speech and non speech stimuli is affected in children with dyslexia. Breier et al [28] observed that children with reading disorder have a deficit in phoneme perception that was evident in inconsistent labeling of tokens in a voice onset tokens (/ga/-/ka/) as well as in their labeling of tone onset tokens, supporting the hypothesis that deficits in speech perception in this group extend to non speech as well as speech stimuli containing similar acoustic cues. The duration between the burst and the onset of voicing is a primary cue for voiced-voiceless differentiation. Thus it can be hypothesized that difficulty in voiced-voiceless differentiation observed in children with learning disorder is due to difficulty in discrimination of durational cues. Furthermore, there is variability within a group of children with dyslexia. Some may have impairment in the processing of speech contrasts whereas others may have impairment in both speech as well as tonal contrasts. This could be because different diagnostic subgroups of dyslexics have different patterns of auditory processing deficits [29]. In the Figure 3, we can notice that MMN is prolonged for /tΣa/-/dZa/, /tΣa/-/sa/, /da/-/δ8a/ and tonal frequency contrasts, while was normal for /da/-/da_s_/ and tonal duration contrasts.

## Conclusion

The present findings support the hypothesis of a basic non linguistic auditory-information processing deficit in individuals with dyslexia, which is also manifested in the preattentive analysis of acoustic features. Some studies have suggested that MMN is a necessary but not a sufficient component for conscious perception of a stimulus change [30]. However, it has been documented that some individuals do not have MMN despite behavioral perception of the stimulus change used to elicit the representation, suggesting that conscious perception of acoustic stimulus differences may not require whatever processes are responsible for MMN generation [31]. It is important to realize that MMN and behavioral responses to same signals represent different aspects of signal processing, the former being preattentive and neurobiological, whereas the latter involves the conscious integration of perceptual information. Nevertheless, biological and perceptual processes that govern how we hear speech may be better understood by understanding how pre-attentive neural processes and conscious perception are related to one another [32].

Thus, the results indicate that discrimination of both speech and non speech stimuli is affected in children with dyslexia speaking Kannada, an Indian language. The findings support the hypothesis of a basic non-linguistic auditory information processing deficit in dyslexic children, which is also manifested in the pre-attentive analysis of acoustic features. The basis of speech discrimination deficits lie in deficits of neurophysiologic encoding along the auditory pathway. Inspite of having good phoneme grapheme correspondence in Indian languages, children who learn these languages do possess impairment in phonological discrimination. Having known the phonological rules used in Indian languages, one would expect lesser processing deficits atleast in the temporal processing. However, temporal processing and spectral processing were affected to equal extent. The result does not provide support to the notion that phonological rules influence the auditory processing. Thus there is a definite need for early identification and rehabilitation of auditory processing disorder in Indian children with dyslexia and MMN can be a valuable tool in the process.

## List of abbreviations

MMN-Mismatch negativity, VOT-Voice onset time.

## Competing interests

The author(s) declare that they have no competing interests.

## Authors' contributions

Both the authors 1) have made substantial contributions to conception and design, or acquisition of data, or analysis and interpretation of data; 2) have been involved in drafting the manuscript or revising it critically for important intellectual content; and 3) have given final approval of the version to be published.

## Supplementary Material

Additional file 1 Audio file of stimulus /tΣa/. This audio file of 250 ms has an unvoiced-palatal-affricate along with vowel /a/ and was used as standard stimulus to elicit MMN. Click here for file

Additional file 2 Audio file of stimulus /dZa/. This audio file of 250 ms has a voiced-palatal-affricate along with vowel /a/ and was used as deviant stimulus to elicit MMN. Click here for file

Additional file 3 Audio file of stimulus /sa/. This audio file of 250 ms has an unvoiced fricative along with vowel /a/ and was used as deviant stimulus to elicit MMN. Click here for file

Additional file 4 Audio file of stimulus /da/. This audio file of 250 ms has a voiced-palatal-stop along with vowel /a/ and was used as standard stimulus to elicit MMN. Click here for file

Additional file 5 Audio file of stimulus /δ8a/. This audio file of 250 ms has a voiced-alveolar-stop along with vowel /a/ and was used as deviant stimulus to elicit MMN. Click here for file

Additional file 6 Audio file of stimulus /da_s_/. This audio file of 175 ms has a voiced-palatal-stop along with vowel /a/ and was used as deviant stimulus to elicit MMN. Click here for file
